# Evaluating the application of failure mode and effects analysis technique in hospital wards: a systematic review 

**DOI:** 10.5249/jivr.v9i1.794

**Published:** 2017-01

**Authors:** Hoori Asgari Dastjerdi, Elahe Khorasani, Mohammad Hossein Yarmohammadian, Mahdiye Sadat Ahmadzade

**Affiliations:** ^*a*^Social Security organization, Isfahan University of Medical Sciences, Isfahan, Iran.; ^*b*^School of Pharmacy, Students’ Scientific Research Center, Tehran University of Medical Sciences, Tehran, Iran.; ^*c*^Health Management and Economic Research Center, Isfahan University of Medical Sciences, Isfahan, Iran.; ^*d*^Shiraz University of Medical Sciences, Shiraz, Iran.

**Keywords:** Failure mode and effects, Risk, Hospital, Systematic review

## Abstract

**Background:**

Medical errors are one of the greatest problems in any healthcare systems. The best way to prevent such problems is errors identification and their roots. Failure Mode and Effects Analysis (FMEA) technique is a prospective risk analysis method. This study is a review of risk analysis using FMEA technique in different hospital wards and departments.

**Methods:**

This paper has systematically investigated the available databases. After selecting inclusion and exclusion criteria, the related studies were found. This selection was made in two steps. First, the abstracts and titles were investigated by the researchers and, after omitting papers which did not meet the inclusion criteria, 22 papers were finally selected and the text was thoroughly examined. At the end, the results were obtained.

**Results:**

The examined papers had focused mostly on the process and had been conducted in the pediatric wards and radiology departments, and most participants were nursing staffs. Many of these papers attempted to express almost all the steps of model implementation; and after implementing the strategies and interventions, the Risk Priority Number (RPN) was calculated to determine the degree of the technique’s effect. However, these papers have paid less attention to the identification of risk effects.

**Conclusions:**

The study revealed that a small number of studies had failed to show the FMEA technique effects. In general, however, most of the studies recommended this technique and had considered it a useful and efficient method in reducing the number of risks and improving service quality.

## Introduction

Safety is the universal concern of all fields in healthcare services. People are likely to suffer from heavy financial losses while receiving unsafe services. Although there is a considerable progress in enhancing patients' safety, there are still numerous drawbacks and damages to the patients caused by side-effects treatment process. ^1^

Clinical risk analysis is one of the essential tasks of hospital managers worldwide.^2,3^ Risk reduction enhances the healthcare services quality, effective relation between hospital staff and patients, and finally it will limit lawsuits for malpractice. Risk management is the ability to identify the existing factors for risk on the one hand, and risks analysis and appropriate strategy selection for controlling and eliminating them on the other hand. ^4,7^ Quality of clinical services is always viewed from different perspectives such as safety, acceptability, and reliability. Acceptability refers to the rate of health service risks acceptance by patients, physicians or other health related staffs who are exposed to those risks. Reliability of a system refers to the possibility of the system’s satisfactory performance under certain working conditions for a fixed period of time. ^8,9^

Medical errors and adverse events are one of the greatest challenges of health systems at the international level. ^9,11^ Approximately, one out of every ten patients referring to hospitals experiences adverse events, which approximately 50% of them are preventable and one-third harms patients. These harms vary from prolonging the patients’ stay to death. ^12^ In general, there are two approaches to investigate human errors in service delivery system of hospitals: personal and systematic approaches. In the personal approach, the focus is on human errors, and people with malpractices are always considered agents of adverse events. In contrast, the systematic approach focuses on the conditions where the fallible man is working. The systems approach assumes that errors are inevitable even in high level and well-known organizations. Hence, according to the systems approach, the best way to tackle errors is to optimize the systems and working processes for humans. ^8,13^

The best way to prevent medical errors is to identify errors and their systemic causes, then learning from them, and finally alerting the system to prevent their reoccurrence.^8^ In this regard, the prospective risk analysis technique is one of the famous risk management and analysis tools which follows the prospective approach based on group risk analysis and attempts to prevent potential errors. Although these techniques are originated form industry, nowadays they are used both in healthcare and industry. ^14^ The Failure Mode and Effects Analysis (FMEA) is one of these techniques. ^15^

FMEA is used to identify potential risks and can be implemented to enhance the patients’ safety.^16^ This systematic method is based on team work for identification, evaluation, prevention, control or the elimination of the causes and effects of potential risks in a system before a final product is delivered to a final user.^8^ Nuclear energy and meteorology and their application in the evaluation and enhancement of complex healthcare safety processes such as intravenous nutrition, medication processes, blood transfusion, and transplantation have increased. Today, Joint Commissions (Accreditation, Health Care and Certification Organization) recommend this model as an introspective risk management model for organizations providing healthcare services in the US. ^17^

The model structure and stages include: forming a team of experts, determining the process and identifying conditions, failure modes and their effects, determining the probability of failure occurrence, severity of effects, and probability of potentials for both failure and effects before the patients or the staff are harmed. Repeatability, severity and identification probability receive a score between 1 to 10, and at the end, a Risk Priority Number (RPN) which is equal to their multiplication is determined and finally this number will be recalculated after the implementation of corrective strategies in order to determine the effectiveness of the model. ^18^ The present study attempted to review unveiled risks by the FMEA technique in various hospital wards. It also tried to identify main aspects of the FMEA model in the hospital wards which include target equipment or process, steps of implementing the FMEA model, the cooperating team, the riskiest and safest activities, and the final impact of model implementation. 

## Methods

This study was systematically conducted in December 2014. In order to find the related articles, the keywords ‘FMEA’, ‘hospital’ and ‘healthcare’ were searched in PubMed, Springer, Science Direct, Google Scholar, Ovid, and Elsevier databases. A total number of 80 papers were found. After reading the papers and eliminating the repetitive ones, 22 papers remained. The followings were the inclusion criteria: 1) the paper must be in English. 2) The paper must have mentioned the FMEA method, 3) The FMEA method must have been implemented in the processes of providing healthcare or medical equipment in a hospital, 5) The paper’s publication date must be after 2010. 

The followings are the exclusion criteria: 1) Papers which had used the FMEA method in producing medications were excluded; 2) Papers which had used a combination of FMEA method with other methods were excluded, too. 

To gather the data, three researchers looked thoroughly into the mentioned databases. Afterwards, two steps were taken to determine the eligibility of papers according to the inclusion criteria. In the first step, two individuals separately studied titles and abstracts of the papers, and after eliminating those papers which did not meet the inclusion criteria, 22 papers were finally selected. In the second step, the whole texts of the papers were separately investigated by two researchers. Then, the data were extracted by the researchers as shown in Table 1. Then, they were investigated to determine the final data. Accordingly, each researcher separately investigated the texts. In case of any disagreement, the researchers discussed the issues. The collected data from the papers included: names of the authors, country/city, year of publication, target equipment or process, steps of FMEA model implementation, cooperating team, the riskiest and safest activities, study objectives, model’s implementation method, and the final impact of the model implementation.

**Table 1:** Summary of articles conducted on FMEA (• Tick means “element reported” • the cross means “element not reported”)

	Authors	Country/City	Publication year	Process/Target equipment	Reporting FMEA steps	Facility	Participating team	Riskiest activities	Safest activity
1	Bagnasco A, et al.	Italy/ Genoa	2013	The process of the relationship among specialized staff	√ Identifying failure modes Identifying effects √ Identifying potential causes of failure Calculating RPN √ Presenting corrective actions	Pediatric emergency in teaching hospitals	Nurses ,physicians, health care support workers	Lack of proper communication with and training of recipients of services	Communication at the time of evaluation for discharge
2	Castello FV, Maher A, Cable G	USA/New Jersey	2011	The process of parenteral nutrition in children	√ Identifying failure modes Identifying effects × Identifying potential causes of failure Calculating RPN √ Presenting corrective actions	pediatric wards	Pharmacists, Nurses, medical technicians, therapists , Nursing educators	-	-
3	Bonfant G, et al.	Italy/ Aosta	2010	Process	× Identifying failure modes Identifying effects √ Identifying potential causes of failure Calculating RPN √ Presenting corrective actions	dialysis units	-	The professional preparation stage	Consumed materials
4	Rosen M. A, et al.	Sierra Leone /Free Town	2014	Process and equipment	× Identifying failure modes Identifying effects × Identifying potential causes of failure Calculating RPN √ Presenting corrective actions	Maternity and trauma hospitals	Anesthetic Nurses	-	-
5	Liao CJ, Ho CC	Taiwan	2014	Process	√ Identifying failure modes Identifying effects √ Identifying potential causes of failure Calculating RPN √ Presenting corrective actions	Hospitals	-	Storage location	Sharp waste
6	Ashley L ,et al.	UK	2011	Process	× Identifying failure modes Identifying effects × Identifying potential causes of failure Calculating RPN √ Presenting corrective actions	Hospitals and outpatient oncology wards	nurses led by an interdiscipli-nary team including administrative staff and researchers of patients’ immunity	-	-
7	Abike F, et al.	Turkey	2010	Process	× Identifying failure modes Identifying effects √ Identifying potential causes of failure Calculating RPN √ Presenting corrective actions	Hospitals	Nurses specialized in pediatrics and obstetrics and gynecology quality	Mothers who have received pain medication and stand without help while their children are in their arms.	the patient Preparation for care delivery on admission
8	Lago P, et al.	Italy/Padua	2012	Process	× Identifying failure modes Identifying effects √ Identifying potential causes of failure Calculating RPN √ Presenting corrective actions	Hospitals/ pediatric wards	-	Children monitoring at the time of drug injection in neonatal intensive care unit (NICU)	Preparation of the medication in pediatric intensive care unit (PICU)
9	Thornton E, et al.	USA/Boston	2010	Process	× Identifying failure modes Identifying effects √ Identifying potential causes of failure Calculating RPN × Presenting corrective actions	Radiology units	-	Losing requests	The lack of nurses for patient transport
10	Lu Y, et al.	China	2013	Process	√ Identifying failure modes Identifying effects √ Identifying potential causes of failure Calculating RPN √ Presenting corrective actions	Blood transfusion units	An improvement quality team led by the manager of the blood transfusion unit including medical staff, nurses and IT staff as well as representatives of logistics and the field of quality control	Insufficient evaluation of requests at the time of blood transfusion and at the time of preparing before injection more than 30 minutes	Lack of testing before transfusion
11	Han TH, et al.	South Korea	2012	Determining blood types	√ Identifying failure modes Identifying risk effects √ Identifying potential causes of failure Calculating RPN √ Presenting corrective actions	Blood banks	Laboratory workers	Errors in test tube labels	An error in the way of shaking a test tube
12	Denny DS, et al.	USA	2014	The radiotherapy process	√ Identifying failure modes Identifying effects √ Identifying potential causes of failure Calculating RPN √ Presenting corrective actions	Oncological hospitals	Oncologist and Nurses specialized in radiation	Physicians’ symptomatic treatment	Treating the wrong patient
13	Weingart SN, et al.	USA/Boston	2011	Drugging process in patients after discharge	× Identifying failure modes Identifying effects √ Identifying potential causes of failure Calculating RPN √ Presenting corrective actions	Oncological clinics	Doctors, nurses, pharmacists, medical technicians, patients and their families, those specialized in the FMEA model, research clinics and nursing research centers	Errors in prescribing, dosage, frequency, delivery of the wrong drug	
14	Walsh KE, et al.	USA,Southwestern USAand Northwestern USA	2012	The process of medication	√ Identifying failure modes Identifying effects √ Identifying potential causes of failure Calculating RPN √ Presenting corrective actions	Pediatric oncology clinics	patients and the patients’ family members as well as pediatricians	The stage of change in the dosage and the lack of training parents at this stage, and disconnection of parents with clinics for follow-ups	-
15	Mesa AF, et al.	Spain	2014	Process	√ Identifying failure modes Identifying effects √ Identifying potential causes of failure Calculating RPN √ Presenting corrective actions	Operating room	Surgeons	Biopsy	Fixing patients on the surgical bed
16	Eijk AC, et al.	The Netherlands / Rotterdam	2013	Process and equipment	√ Identifying failure modes Identifying effects √ Identifying potential causes of failure Calculating RPN √ Presenting corrective actions	NICU	Neonatologists, Professional nurses, ICU Nurses,Instructor of Nursing, PhD, students	Slow decline in fio2	No change of alarm scope by nurses
17	Funk KH, et al.	USA	2010	Process	× Identifying failure modes Identifying effects × Identifying potential causes of failure Calculating RPN √ Presenting corrective actions	Operating room	Surgeons and surgical assistants, engineers of industries	-	-
18	Perks JR, et al.	USA/California	2011	Radiation therapy process	√ Identifying failure modes Identifying effects √ Identifying potential causes of failure Calculating RPN × Presenting corrective actions	Radiotherapy Clinic	Medical physicists,radiologists, oncologists, Head of the Radiotherapy Center, The secretory of the Office of Quality Improvement	Using inappropriate laser	Patient movement during treatment
19	Noel CE, et al.	USA	2014	Equipment	× Identifying failure modes Identifying effects √ Identifying potential causes of failure Calculating RPN √ Presenting corrective actions	Radiotherapy Clinic	Medical physicists,Oncologists, radiotherapists medical engineers	-	-
20	Duncan JR, et al.	USA/Washington	2010	Central catheter insertion process	× Identifying failure modes Identifying effects × Identifying potential causes of failure Calculating RPN √ Presenting corrective actions	-	Specialists in internal medicine, surgery, radiology, emergency medicine, anesthesiology, neurology, gynecology, professional nurses, and nurses of intensive care unit (ICU)	-	-
21	Yarmohammadian MH, et al.	Iran/Isfahan	2014	Process	√ Identifying failure modes Identifying effects √ Identifying potential causes of failure Calculating RPN √ Presenting corrective actions	ICU	Nurses of intensive care unit	-	-
22	Jabbari A, et al.	Iran/Isfahan	2014	Process	× Identifying failure modes Identifying effects √ Identifying potential causes of failure Calculating RPN √ Presenting corrective actions	Operating room	Operating room technicians, anesthetic nurses and technicians and health workers	-	-

## Results

The complementary results of the study are presented in Table 1. Regarding the topic of implementing the model, 19 studies had focused on the processes, one was on medical equipment, and 2 studies had focused both on medical equipment and processes. Regarding the levels of implementation of the FMEA model, only 4 papers had covered all the stages, 21 papers had investigated risks phase identification, 12 papers had mentioned the rout case analyze, and only 14 studies had computed the RPN. 

Among those studies that focused on hospital services, 1 of them had been implemented in an Intensive Care Unit (ICU), 3 studies in operating rooms(OP), 2 studies in radiotherapy departments, 5 studies in oncology clinics, 1 study in Neonatal Intensive Care Unit (NICU), 2 studies in blood banks, 1 study in a radiology unit, 2 studies in pediatric wards, 2 studies considered the hospital as a whole, 1 study in maternity and trauma wards, 1 study in a dialysis unit, 1 study in an emergency department, and 1 study in a children’s specialized hospital. 

Considering the model implementation team, 11 studies were conducted by nurses, 3 studies were done by physicians, management and managing staff, quality experts, oncologists, pediatricians, surgeons and surgery assistants; the participants were pharmacists, health staff, medical technicians, medical physicists, head nurses and medical students, safety officials, gynecologists, radiotherapists, patients and their families in 2 studies, and in one study the participants were industrial engineers, medical engineers, anesthesia technicians, operating room technicians, secretaries, neurologists, Information Technology staff, service providers, laboratory staff, radiotherapists, equipment engineers, radiologists, urgency medicine specialists, and internal medicine specialists and finally the studies which concentrated on the risk of activities. The results of the FMEA model demonstrate that 14 studies had identified both low-risk and high-risk activities and 2 studies had identified only the riskiest activity. 

## Discussion

This study was an attempt to examine comprehensively different dimensions of the FMEA model implementation in the field of process and medical equipment in hospital wards.

The first article, by Bagnasco et al., attempted to identify appropriate and effective measures taken for patients' safety in NICU. All the model’s steps were implemented, but the researchers didn't mention the effects of the model implementation. The Situation-background-assessment-recommendation (SBAR) tech-nique was proposed as their strategy. Having imple-mented this model, the researchers had concluded that the greatest number of repeatable and harmful risks was related to inappropriate relationships among the staff, particularly during the patients’ transfer and re-lease.^19^

In the second article by Castello et al., the FMEA model had been used to decrease the infections in central veins due to intravenous nutrition in children. By collecting the data related to quality enhancement processes and benchmarking, the paper attempted to make appropriate guidelines for children’s intravenous nutrition. For implementing the model, first the intravenous nutrition process had been divided into 4 activities: 1) ordering the implementation of Total Parenteral Nutrition (TPN), 2) receiving medications at the drug store, 3) quality control, 4) injections done by the nurses. In the next step, TPN therapy had been divided into three steps: 1) preparation steps before injection such as washing hands and checking a child’s vein, 2) checking connections to the central vein, 3) dressing the central vein’s spot such as: dressing change stages to ensure that the conditions are under control. The results showed that the FMEA implementation reduced the infection resulting from the central vein due to intravenous nutrition from ¾ to ¼.^2020^

The objective of Bonfant et al. in the third article was to identify and reduce risks and increase the patients' safety in a dialysis unit. The implementation included these stages: 1) the process identification, 2) the analysis of risks and determining RPN, 3) planning and forming an S, O, D (severity-occurrence-detectability) matrix and dividing it into 4 areas (high-risk or urgent, force majeure, programming and control), 4) the interventions and 5) implementing the model again. Using this model, the researchers realized that the greatest cause of risks in dialysis unit were miscommunications and organization in the process implementation route; and after implementing the FMEA and corrective interventions such as preparing dialysis instructions and nursing information sheets, the number of failure modes was reduced. ^21^

The Fourth article by Rosen et al. was an attempt to identify risk cases of anesthesia care and to offer strategies to reduce those cases employing FMEA model. This model had been implemented in several stages: 1) identifying and understanding problems, 2) team-building and brainstorming, 3) risk identifying, and 4) brainstorming to reduce the risks. The study was successful in identifying the factors affecting the effectiveness of the anesthesia machine and developing strategies to reduce the risks.^22^

The objective of Liao et al., in the fifth article, was selecting the best way to dispose hospitals medical waste in crisis. The FMEA had been implemented through distributing a questionnaire including following items; accessibility of freezing devices, accessibility safety boxes, and disposal frequency and volume. After implementing FMEA and calculating the RPN, the interventions had been suggested. The paper showed that FMEA had been used to reduce the danger of outsourcing medical waste disposal services, and hospitals could clearly identify and evaluate the risks of biomedical waste. ^23^

The sixth article, by Ashley et al., aimed at identifying the possible situations of chemotherapy failures and suggesting treatment strategies to address those failures. This paper also collected users' feedback in the FMEA process. Using the FMEA method included chemotherapy process mapping, identification and prioritization of possible risks (failure cases) for each stage of treatment strategies. In general, paper 6 reported positive aspects of users' feedback, analysis process, multi-disciplinary teamwork and communications. ^24^

The seventh paper, by Abike et al., tried to develop new scales for risk evaluation and preventive measures for newborn babies fall from admission to discharge. Different steps of FMEA were taken. Results showed that the number of falls as well as RPN had been reduced after the corrective interventions.^25^

The eighth article by Lago et al. examined the identification of risks in kids’ injection process. This will enhance a patient's safety during an injection. In implementing FMEA, a multidisciplinary team was formed. The time was a limitation for this study and there was a need for individuals who had knowledge and were involved in the process.^26^

The ninth article, by Thornton et al., attempted to investigate the related equipment and processes in a clinical radiology department. First of all, the process of MRI scan (Magnetic Resonance Imaging) had been divided into sub-processes, and then the risk had been identified and scored. Implementing this model helps emphasize the risks of sub-processes, reduce their future occurrence, enhances patients' safety and increases productivity in the radiology department. The implementation included forming a team, processes identifying, process map designing, identifying and scoring risks, and determining the results. The results demonstrated that the use of FMEA is effective in identifying risks after the use of a new smart pump in the medication process.^27^

The objective of Lu et al., in the tenth article, was to manage blood transfusion risks, improve blood component quality, and ensure patients' safety. The steps of implementing FMEA included: forming a team, dividing the blood transfusion process into sub-processes, scoring the risks, and introducing corrective interventions. Finally, the results showed that this technique is a useful instrument for an active analysis and reducing blood transfusion risks.^28^

The 11th article, by Han et al., attempted to use the FMEA model to compare potential risks of blood type determination both manually and automatically. To implement the FMEA, first six laboratories had been selected across South Korea and the target staff received the needed training. Afterwards, their blood type had been determined both manually and automatically. Then, the process had been divided into five steps and each step had been divided into sub-steps, causes and effects of risks, interventions and RPN of each sub-process had been identified. Finally, these two methods (manual and automatic) were compared. Using this model, the authors were able to highlight the potential risks of the manual method and concluded that using the automatic method substantially reduces the risks level, so it’s more effective despite its costs.^29^

The objective of Denny et al., in the 12th article, was the use of prospective risk management model in the radiotherapy of patients who suffer from cancer, and tried to establish a national network for all oncology specialized hospitals. They followed these steps: selecting a process and determining the sub-processes, forming specialized teams, drawing process map, analyzing risks, implementing the process completely and measuring its consequences. Using FMEA, the radiotherapy process for patients with cancer was evaluated, the risks were identified, and the model was developed and implemented to avoid these risks.^30^

The purpose of Weingart et al., in 13th article was using introspective risk analysis in giving oral medications to outpatients suffering from cancer, identifying risk cases and omitting them. FMEA was implemented in the following steps: selecting five most frequently used medications by outpatients, forming a team, developing a process map for each medication, identifying risk cases for each medication separately, selecting the riskiest error cases, giving advices to reduce risk, comparing risks in terms of their severity. The results showed that after comparing the medications and determining risks and their causes the following suggestions were offered: preparing pamphlets and policy and procedures for both patients and nurses, practical guidelines for physicians and finally developing patients' follow up plans. Using this model, researchers identified the complexities of outpatients' medication process and developed a comprehensive method to reduce risks.^31^

The 14th article, by Walsh et al., attempted to use a risk management model to identify risks in children with cancer who received in-home care from their parents and offer appropriate strategies to reduce the number of failure modes. Implementing FMEA included the following steps: choosing three outpatient oncology clinics, choosing a group of English speaker parents, introducing FMEA to them, brainstorming, choosing the riskiest activity, conducting an evaluation by pediatricians, taking 13 corrective steps for high-risk activities, developing strategies and interventions such as the use of emails and the continuous communication with parents or sending nurses to their homes and providing more training to enhance their information about medications that are used by children. The researchers developed a better understanding of these children’s problems and realized the parents’ willing to participate in the implementation of the FMEA model.^15^

The 15th article, by Mesa et al., was an attempt to improve surgeons’ skills and patients' safety in laparoscopy. The FMEA implementation included the following steps: forming a team of 48 surgeons, dividing them into 24 teams of 2, training on laparoscopy by examining laboratory animals in three steps and finally analyzing the results. The results demonstrated that, at the end of the third stage, the RPN score of all groups except for two groups (due to their low technical aptitude) decreased gradually. In general, the findings revealed that the use of FMEA principles in laparoscopy training could enhance the surgeons’ nontechnical skills.^32^

The objective of van der Eijk et al., in the 16th article, was to investigate and identify the potential threats in an oxygen therapy process with complementary devices in premature infants hospitalized at Notre Dame Hospital in the Netherlands. The FMEA implementation included the following steps: defining the subject or the process, forming a multi-disciplinary/ multi- specialty team, dividing the process to sub-processes, analyzing the risk (determining the degree, severity of effects, offering strategies, giving RPN, and identifying 10 high-risk activities) and developing strategies for eliminating these risks. Using FMEA, the researcher has identified potential risks in the oxygen therapy of premature infants who receive oxygen from complementary devices and developed strategies.^33^

The 17th article, by Funk et al., attempted to identify human failures in the laparoscopy process. The FMEA implementation included the following steps: forming a team, identifying the process, designing the process map, identifying the risks and scoring them, and determining the results. The results provide a base for the application of both medical and human factors engineering perspectives to yield a comprehensive list of vulnerabilities to human errors and factors that may cause them.^34^

The objective of Perks et al., in the 18th article, had been to increase patients' safety in a radiotherapy process. The FMEA implementation included the following steps: determining the patients’ receiving steps, physicians’ checking step, determining the process flowchart, scoring the risks, determining RPN, focusing on high-risk activities and developing strategies to reduce them. The results showed that the use of this model helped develop strategies to enhance the patients' safety.^35^

The 19th article, by Noel et al., attempted to conduct an investigation on comparative radiotherapy barriers by using the FMEA model. The FMEA implementation included the following steps: comparing both IMRT (Intensity Modulated Radiation Therapy) as a treatment delivery technique and ART (Adaptive Radio Therapy) as a method for treatment in radiotherapy, determining the activities, determining risks, scoring the RPN, and developing strategies. They analyzed some specific risks for both IMRT and ART. They concluded that improving a method adds new risks which can also be evaluated and mitigated.^36^

The 20th article, by Duncan et al., attempted to use the FMEA for developing a formal and standard curriculum about the replacing and taking care of central catheters for medical students. The FMEA implementation included the following steps: three years of counseling, training 124 doctors and 6 nurses in 9 wards, and training newly-graduated interns on placing central catheters, enhancing accessibility by using ultrasound, and training how to use sterile sets to prevent infections. Using this model, the researchers identified the complexities related to the process of replacing the central catheter. Although the infections related to this process have not been reduced yet, the researchers succeeded in enhancing the safety during catheter replacement by identifying and controlling the implementation of the process.^37^

The 21st article, by Yarmohammadian et al., was an attempt to identify and evaluate potential risks in an ICU. The researchers used FMEA standard worksheets to analyze potential risks and their effects. Key activities were selected by the nurses, then during several sessions the potential risks, their causes and effect had been identified. Based on verified standard scales, their degree of severity, occurrence, and detectability had been determined through brainstorming, and the RPN number had been computed for each potential case.^2^

The twenty-second article by Jabbari et al. attempted to identify, assess and provide appropriate actions to control, reduce and eliminate the potential risks in operating rooms for eye surgery at a hospital. Based on the authorities’ point of view, eight main actions had been selected to analyze the potential risks. 35 failure modes had been identified in the operating rooms. The study scored three criteria named severity, occurrence, and detection.^38^

## Conclusion

The results of the studies showed that this model has been mostly implemented in Pediatric and Oncology wards and most of the studies have focused on the processes. Most of the papers have tried to mention all the steps of the model, and after implementing the corrective strategies and interventions, they have recalculated RPN to determine the model's effectiveness. However, they have paid little attention to identifying the risk effects. In general, most studies have recommended this model and have considered it a useful instrument for decreasing the number of risk elements and enhancing the quality of services.
